# Inorganic Polyphosphate—Regulator of Cellular Metabolism in Homeostasis and Disease

**DOI:** 10.3390/biomedicines10040913

**Published:** 2022-04-15

**Authors:** Filip Kus, Ryszard T. Smolenski, Marta Tomczyk

**Affiliations:** 1 Laboratory of Molecular Biology, Intercollegiate Faculty of Biotechnology of University of Gdansk and Medical University of Gdansk, 80-307 Gdansk, Poland; kusfi@gumed.edu.pl; 2 Department of Biochemistry, Medical University of Gdansk, 80-211 Gdansk, Poland

**Keywords:** inorganic polyphosphate, inflammation, neurodegenerative diseases, SARS-CoV-2, cancer

## Abstract

Inorganic polyphosphate (polyP), a simple anionic polymer consisting of even hundreds of orthophosphate units, is a universal molecule present in both simple and complex organisms. PolyP controls homeostatic processes in animals, such as blood coagulation, tissue regeneration, and energy metabolism. Furthermore, this polymer is a potent regulator of inflammation and influences host immune response in bacterial and viral infections. Disturbed polyP systems have been related to several pathological conditions, including neurodegeneration, cardiovascular disorders, and cancer, but we lack a full understanding of polyP biogenesis and mechanistic insights into the pathways through which polyP may act. This review summarizes recent studies that describe the role of polyP in cell homeostasis and show how disturbances in polyP levels may lead to disease. Based on the collected findings, we highlight the possible usage of this polymer as a promising therapeutic tool in multiple pathologies.

## 1. Introduction

Inorganic polyphosphate (polyP) is a linear polymer of orthophosphate units covalently linked by high-energy phosphoanhydride bonds, as in adenosine triphosphate (ATP). As an anionic polymer, it carries a negative charge at physiological pH [1]. PolyP is a highly conserved molecule, present in organisms across all living systems, including archaea, bacteria, fungi, plants, and animals [2].

Despite being structurally very simple, the length of the polymer varies across organisms, which often serves as a distinguishing feature. Bacteria produce long-chain polyP that comprises up to 1000 phosphate residues or more, often in the form of large granules called acidocalcisomes. Eukaryotic cells synthesize shorter polyP chains ranging from around 80 P_i_ units in human platelets to 200 P_i_ residues in yeast [3]. The enzymes responsible for polyP synthesis in higher eukaryotes have not been fully identified yet [4], as the synthesis of this polymer has been extensively studied mostly in bacteria. Key prokaryotic enzymes involved in polyP metabolism include polyphosphate kinase (PPK), which catalyzes the synthesis of polyP using ATP as a phosphate donor, and exopolyphosphatase (PPX), responsible for polyP degradation to free P_i_ [5]. Some bacteria, such as *Escherichia coli* possess one PPK enzyme, others possess two homologs: PPK1 and PPK2 (e.g., *Pseudomonas aeruginosa*, *Francisella tularensis*); and some can have neither [6,7]. Interestingly, homologs of PPK have not been found in higher eukaryotes [8].

The physiological role of polyP in bacterial cells has for years been linked to stress response, phosphate storage, and, more recently, protein folding [9,10,11]. Bacteria elevate polyP synthesis in response to environmental stress conditions, such as amino acid starvation or oxidative stress [12,13]. It has been shown that under stress conditions polyP plays a crucial role in the regulation of bacterial DNA replication [14]. Gross and Konieczny observed inducible proteolysis of replication initiator protein DnaA by Lon protease in the presence of polyP in a process termed PolyP-induced DnaA proteolysis (PDAP). In mutant *Escherichia coli* cells that lack PPK enzymes (*E. coli* Δ*ppk*) and are therefore not able to synthesize polyP, DnaA protein levels remained stable after stress induction [15].

In addition to its principal role in the regulation of stress response, bacterial polyP has been linked to a variety of other functions. Pathogenic bacteria mutants unable to synthesize polyP were defective in motility [16], biofilm formation [17], cell signaling, and production of virulence factors [18], which suggests polyP’s importance for proper cellular functioning and metabolism.

PolyP’s role in eukaryotic cells is not as well described as in prokaryotes. Nonetheless, there is an increasing amount of research that focuses on elucidating how this ancient molecule shapes eukaryotic cell metabolism, both in homeostatic and pathogenic conditions. Thus, this review highlights open questions and presents polyP as a potent regulator of cellular metabolism, not only in healthy cells but also during infections, tumorigenesis, neurodegeneration, and other pathologies.

## 2. Polyphosphate as a Regulator of Homeostasis in Eukaryotic Cells

PolyP in higher eukaryotes, specifically in mammals, is present in a broad range of tissues. In rodents, it has been found in the brain, heart, kidneys, liver, and lungs [19]. PolyP has been also found in the lysosomes of human fibroblasts, the nucleoli of human myeloma cells, mitochondria, plasma membranes, microsomes, and cytoplasm compartments of various cell types, as well as in the extracellular space, where it can be released by activated platelets and astrocytes [19,20,21,22,23]. PolyP amounts in mammalian cells oscillate in a micromolar range and are considerably lower than those observed in bacteria [24]. The highest concentration of mammalian polyP was described for platelets, where it reaches around 1 mM [25]. High levels were also observed in bone tissue (several hundred μM polyP in osteoblasts) [26].

In the past century, most research has focused on identifying polyP in various mammalian cells, but the role it may serve was only discussed speculatively [20,27,28]. These speculations covered its function as a regulator of lysosomal transmembrane potential, phosphate storage, or as an energy source. The first indications of the substantial regulatory role of polyP in eukaryotic homeostasis have been described by Ruiz and colleagues [25]. They found that granules of human platelets, which are similar to bacterial acidocalcisomes, are rich in polyP that is released upon thrombin stimulation. Smith et al. described how the polyP of platelets exerts an procoagulant effect and triggers a clotting cascade in the presence of factor XII (FXII), and presented polyP as an activator of the contact pathway of blood clotting [29]. The contact pathway (reviewed by Yi Wu [30]) consists of several plasma proteins activated by negatively charged surfaces or anions (like polyP). PolyP binds and activates FXII, triggering the contact pathway, which leads to blood coagulation and proinflammatory response through the production of the bioactive peptide bradykinin. Furthermore, polyP may be incorporated into fibrin and stabilize fibrin clot structure, making it more resistant to fibrinolysis [31]. Procoagulant effects of polyP are also pronounced; it has the ability to inhibit anticoagulant factors such as tissue factor pathway inhibitor (TFPI) released by endothelial cells [32].

In addition to its procoagulant and proinflammatory functions, polyP released from platelets and platelet-rich plasma have been linked with cell proliferation and tissue regeneration. Müller and colleagues showed that polyP promotes the growth and viability of bone marrow-derived mesenchymal stem cells and upregulates the expression of transcription factors responsible for osteogenesis and chondrogenesis, showing the involvement of polyP in bone and cartilage formation/homeostasis [33]. They also showed that calcium–polyP microparticles are taken up by cells via clathrin-dependent endocytosis; thus, polyP in the form of such microparticles can be manufactured and utilized in treatments of osteoarticular pathologies.

**Figure 1:**
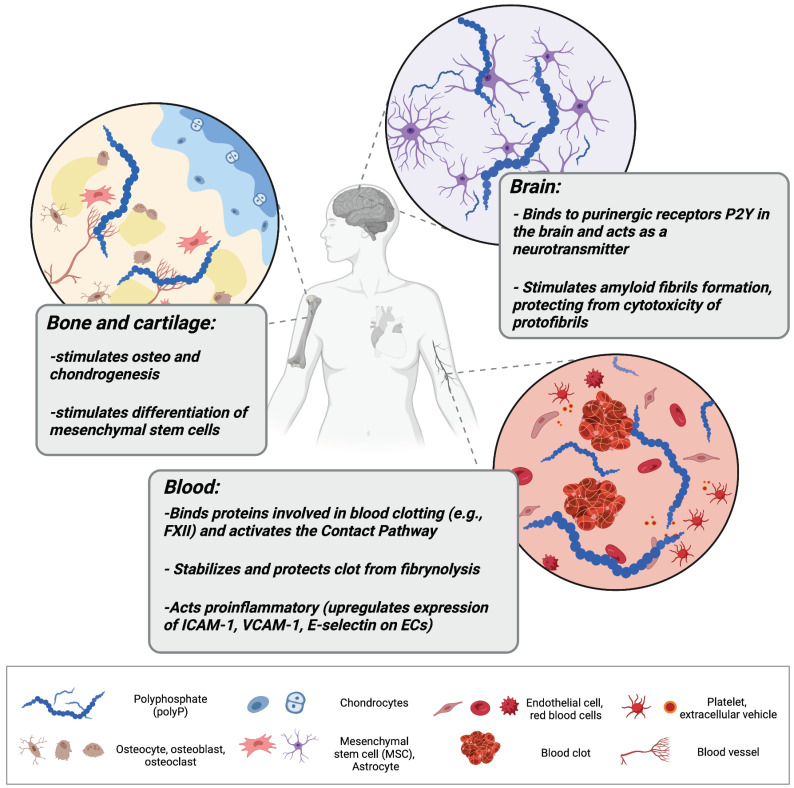
Polyphosphate (polyP) regulates a variety of processes in different tissues in the human organism. In bone tissue, polyP stimulates osteogenesis and chondrogenesis, and promotes the growth and differentiation of bone marrow-derived mesenchymal stem cells (MSCs). In the brainstem, it can be taken up by activated astrocytes and act as a mediator of signal transmission. In the cardiovascular system, polyP can be released within extracellular vehicles by activated platelets, where it activates the contact pathway of blood clotting, stabilizes fibrin clot structure, and mediates proinflammatory responses by activating endothelial cells (ECs).

Interestingly, polyP can act also as a mediator of signal transmission in the mammalian brain. Astrocytes activated via polyP, similarly to Ca^2+^ activation, release endogenous polyP which is further cleared from the extracellular space by neuronal uptake, suggesting that polyP acts as a glio- and neurotransmitter [23,34]. PolyP mediates communication between astrocytes by binding to purinergic receptors P2Y_1_ in the brainstem [23]. P2Y receptors are G protein-coupled receptors, widely distributed within the cells of the human body [35]. They are activated by extracellular nucleotides and mediate a myriad of signaling cascades involved in cell development, proliferation, and also immune regulation and inflammation [36,37]. PolyP binding to astroglial P2Y_1_ results in an increase in central sympathetic activity, stimulates breathing, and raises arterial blood pressure in vivo in rats [23]. Furthermore, several studies have demonstrated polyP as a mediator of proteostasis (reviewed by Xie and Jakob [38]), suggesting a substantial role of polyP in neurodegenerative disorders. Protein aggregation and production of insoluble fibers called amyloid fibrils are the foundation of neuro-diseases such as Alzheimer’s or Parkinson’s diseases. The intermediates preceding mature fibril formation, such as oligomers and protofibrils, accumulate in the extracellular space (and synapses) and alter cell communication, mitochondrial function, eventually triggering apoptosis [39]. PolyP has been shown to act neuroprotectively and abrogate the neurotoxic activity of improperly aggregated amyloid β-peptides/proteins and Tau protein which are responsible for the onset of Alzheimer’s disease [40]. Furthermore, polyP levels have been shown to shrink significantly in the brain with aging [41], when neurodegenerative disorders are most to likely to occur. Figure 1 collects and presents the activity of polyP in different tissues of the human body.

### PolyP in Mitochondrial Homeostasis and Cell Energetics

It is well known that mitochondrial dysfunction might be another critical factor and common feature in neurodegeneration [42]. Recently, Angelova and colleagues showed that approximately 40% of cellular polyP in astrocytes resides in mitochondria [43], where it regulates mitochondrial activity and calcium handling [44]. PolyP in mitochondria acts as a buffering system and prevents the formation of calcium phosphate insoluble precipitates, thus maintaining mitochondrial calcium homeostasis and sustaining high levels of calcium in the bioavailable form [45,46]. Disrupted calcium homeostasis and a decline in mitochondrial function are hallmarks of aging and, in addition to neurodegeneration, have also been associated with coronary heart disease and diabetes [47]. On the other hand, Abramov et al. showed that a depletion of mitochondrial polyP by expression of yeast PPX in several cell lines (including hepatic carcinoma cells, human embryonic kidney cells, and mouse myoblasts) reduces calcium-dependent mitochondrial permeability transition, a key mechanism underlying necrotic and apoptotic cell death [48,49]. Mitochondrial pores are formed upon stressing stimuli and calcium mishandling and also contribute to the process of neurodegeneration in Parkinson’s, Alzheimer’s, and Huntington’s diseases [50]. Similar results were shown in cardiomyocytes, where polyP depletion also leads to the inhibition of mPTPore (mitochondrial permeability pore) formation. Reduction of polyP in cardiac cells may be cardioprotective, as the formation of mPTPore and dysfunction of mitochondria lead to pathologies in cardiac tissue and irreversible cardiac cell injuries [51]. However, research on cardiac myocytes demonstrated a dual role of polyP, which is directly linked to its chain length. While polyP of 14 phosphates activated mPTPore formation, longer polyP molecules (130 phosphates) suppressed mPTP activity [52]. Seidlmayer et al. hypothesized that such competing actions of polyP may stem from polyP’s chaperone activity and ability to bind proteins involved in mPTPore opening. The authors concluded that mitochondrial polyP chain lengths depend on the metabolic state of these organelles, hence the polyP role in mitochondria should be considered in relation to the function of polyP in cell bioenergetics.

Mitochondria are the key energy producers in cells. Interestingly, Pavlov and colleagues described that mitochondrial polyP play an important role in mammalian energetics [53,54]. They observed dynamic changes in polyP levels in astrocytes that were directly triggered by inhibition or activation of mitochondrial respiration. Inhibition of glycolysis by the addition of iodoacetic acid, which blocked the supply of substrates for mitochondrial respiratory complexes, reduced polyP abundance in mitochondria, suggesting that polyP levels may depend on the activity of the respiratory chain. Confirming this observation, in another study, Nakamura and colleagues observed that degradation of polyP enhances lactic acid fermentation in mice expressing the polyP-degrading PPX enzyme [55]. Their model proposes that elongation of polyP and a subsequent reduction in free intracellular P_i_ concentration sustains mitochondrial respiration and suppresses anaerobic lactic acid production. In a recently published study, Abramov and his group showed that ATP synthase, the mitochondrial inner membrane enzyme responsible for the formation of ATP, is involved in polyP synthesis similarly to the synthesis of ATP [56]. Using isolated rat brain mitochondria, they showed that polyP production is blocked in the presence of oligomycin, an ATP synthase inhibitor. Moreover, application of ATP before or after oligomycin did not affect polyP concentration, which excludes the possibility of ATP being an intermediate product of polyP synthesis. ATP synthase can also function in the opposite direction, as a proton pump hydrolyzing ATP. The authors observed that in the absence of ATP polyP can by hydrolyzed by ATP synthase, proving that polyP can be utilized by eukaryotic cells as a direct source of energy.

However, polyP is not only synthesized in mitochondria. Significant amounts of this polymer can be found in other structures, including the secretory granules of platelets or lysosomes of other cell types (e.g., fibroblasts and glial cells) [20,25,43]. These observations suggest that other enzymes, not only the mitochondrial ones, should also be involved in polyP biogenesis. Reusch et al. proposed that a plasma membrane calcium pump (Ca^2+^-ATPase) from erythrocytes functions as a polyphosphate kinase due to its ATP/ADP-polyphosphate transferase activities [57]. Some authors have also suggested that multiple enzyme complexes may be involved in the process of polyP formation and that polyP may be a byproduct of several enzymatic reactions [54]. Clarifying the issue of polyP synthesis in eukaryotic cells or finding enzymes responsible for polyP production in other intracellular locations is important to allow for further advances in polyP studies.

PolyP may not only act as a direct energy source but also as a phosphate store. For instance, in bacteria, both ATP and polyP are important phosphoryl donors for NAD kinase, which utilizes this polymer to yield NADP^+^ from NAD^+^; however, eukaryotic NAD kinases use only ATP, suggesting another purpose of polyP in mammalian phosphate-metabolism [58,59]. Indeed, recently, an interesting concept has emerged, in which polyP is proposed as both an energy and phosphate source in the extracellular space. Purines and their derivatives, ATP, ADP, and adenosine are important signaling molecules that act through purinergic receptors. Nucleotides can be released from cells by microvesicles, membrane channels, and transporters, or dying cells, and the extracellular adenosine is generated via adenine nucleotide hydrolysis by plasma membrane nucleotidases [60]. Müller et al. hypothesized that polyP may also participate in extracellular nucleotide generation. They found increases in extracellular ATP and ADP levels after polyP treatment of human sarcoma osteogenic (Saos-2) cells [61]. Moreover, they underlined that incubation of Saos-2 cells with polyP leads to translocation of alkaline phosphatase (ALP) and adenylate kinase (AK) to the cell membrane and further release of these enzymes outside of the cell in matrix vehicles. Both of these enzymes are involved in the interconversion and dephosphorylation of extracellular nucleotides. The increase in the ATP pool after polyP stimulation can both be utilized in purinergic signaling or as an energy reservoir, especially in tissues that consist of a large extracellular matrix in which only a few cells are embedded (e.g., bone and cartilage) [53]. It would be interesting to further investigate how polyP influences the extracellular purinergic system or whether it acts through purinergic receptors in other tissues. It is well known that disruption to purinergic signaling contributes to the pathophysiologies of multiple disorders in the immune system, vasculature, heart, kidneys, lungs, and the brain [62]. Nevertheless, polyP influence has not been investigated in the context of Huntington’s disease, a multi-system disorder which comprises both malfunction of purinergic signaling and mitochondrial dysfunction [63]— crucial polyP-associated metabolic events.

**Figure 2:**
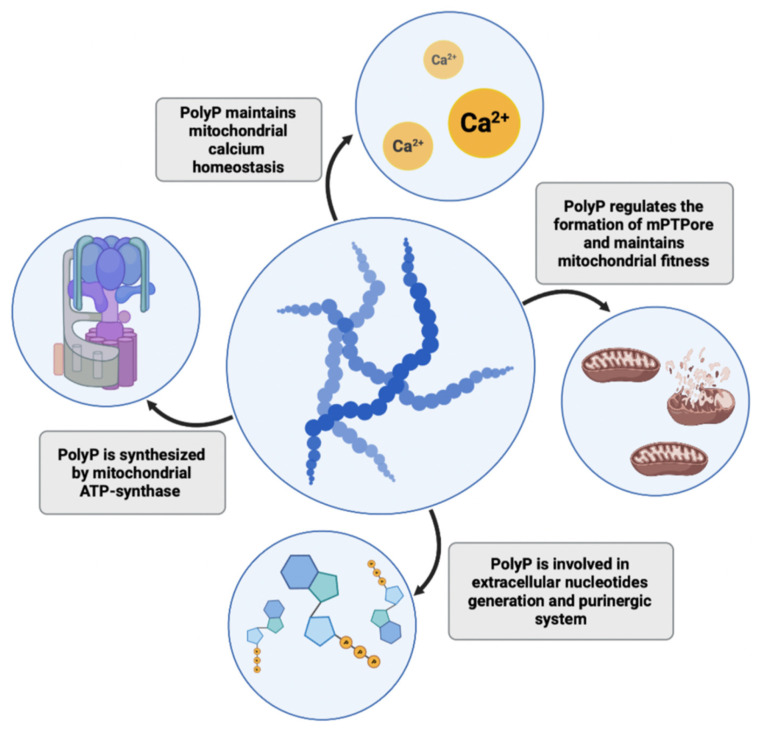
PolyP plays several roles in mitochondria and cell bioenergetics. The polymer is synthesized in mitochondria by ATP synthase, where it maintains calcium homeostasis and protects against the formation of calcium phosphate precipitates. It also regulates the processes of mitochondrial permeability transition and formation of mitochondrial permeability pores (mPTPores), thus maintaining mitochondrial fitness. PolyP is also involved in the purinergic system and the generation of extracellular nucleotides.

The collected findings highlight polyP as a multifunctional molecule that plays a key role in maintaining proper cellular homeostasis; thus, deteriorations in its intra- or extracellular levels may lead to the development of multiple pathologies. PolyP functions in mitochondrial homeostasis and energetics are presented in Figure 2.

## 3. Regulatory Role of PolyP in Infection and Inflammation

During infection, bacterial cells must withstand various environmental stresses, including changes in temperature, pH, or exposure to different components of the innate immune system, e.g., antimicrobial peptides [64]. Interestingly, it has been found that factors such as oxidative stress and nutrient limitations may upregulate bacterial polyP synthesis [13,65]. Moreover, Roewe et al. have recently demonstrated that the severity of sepsis induced by *E. coli* infection varies depending on whether bacteria can or cannot synthesize polyP [66]. Mice infected with a wild-type *E. coli* strain capable of polyP synthesis displayed poor survival rates. Survival improved in mice infected with an *E. coli* PPK-deficient strain and those treated with PPX, an enzyme that degrades polyP. Furthermore, polyP effects appear to be chain length-dependent. When authors injected bacteria together with chemically synthesized long-chain polyP (which resembles bacterial polyP) into the peritoneal cavities of mice, accelerated mortality was observed, while no significant difference in mortality was found with co-injection of short-chain polyP and bacteria. The difference in the course of sepsis was a result of a weakened myeloid cell response, in particular, an impaired macrophage phagocytosis of bacteria co-injected with long-chain polyP.

Macrophages are the key components in host defense against bacterial infection. One of the classification systems groups these cells into two subpopulations: M1 and M2 macrophages. Polarization to the M1 phenotype, the *Classically Activated Macrophages,* can be driven by LPS and stimulates host defense response to infection. These macrophages are characterized by high antigen presentation, high expression of proinflammatory cytokines, and higher production of reactive nitrogen or oxygen intermediates [67]. Roewe’s study showed that long-chain polyP can be internalized by macrophages and misdirect their polarization towards the M2 phenotype, the *Alternatively Activated Macrophages* that play a role in wound-healing and display immunosuppressive features [68]. Macrophages incubated with long-chain polyP showed lower transcription levels of M1 phenotype-associated genes, such as *iNOS*, the transcript of which encodes inducible nitric oxide synthase, which exerts a cytotoxic effect on microorganisms during infection [69], or *CXCL10*, encoding a macrophage-attracting chemokine. Long-chain polyP, but not the short-chain, induced CD206 protein expression, the marker of the M2 phenotype. Moreover, long-chain polyP suppressed the transcript levels of MHC-inducing transcription factors and subsequently reduced MHCII and costimulatory proteins CD80 and CD86 expression, influencing macrophages’ antigen-presenting capacities (this effect, again, was not observed with short-chain polyP). In fact, long-chain polyP modulated the expression of more than 1800 genes regulated by LPS/TLR4 signaling and suppressed the expression of hundreds of interferon-regulated genes in macrophages. In conclusion, the production of polyP may be an evasion strategy for bacteria, allowing them to escape from host innate immune responses.

The immunosuppressive actions of long-chain polyP are surprising when compared to the often documented proinflammatory role of short-chain polyP [70,71,72]. Chrysanthopoulou et al. recently reported that short polyP activates neutrophils and stimulates the release of neutrophil extracellular traps (NETs), which are known extracellular structures that can trap, neutralize, and kill bacteria [73,74]. Studies on endothelial cells such as HUVECs demonstrated that platelet-like polyP (with a length of around 70 P_i_) enhances the barrier permeability of endothelial cells (ECs) and stimulates the expression of adhesion molecules, such as VCAM-1, ICAM-1, and E-selectin [71]. These molecules are engaged in leukocyte recruitment and binding to ECs and their expression is up-regulated in response to proinflammatory stimuli, such as cytokines or endotoxins [75]. Moreover, short-chain polyP has been shown to amplify proinflammatory responses by binding to histone H4 and high mobility group box 1 (HMGB1) proteins, which are late mediators of inflammation. PolyP together with H4 and HMBG1 activates pro-inflammatory signaling pathways through the EC surface receptors RAGE and P2Y_1_ and the subsequent NF-κB pathway [70]. In a recently published follow-up study, Rezaie’s laboratory demonstrated that platelet-like polyP together with HMGB1 can also induce von Willebrand factor (VWF) release from endothelial cells; however, the consequences of this interaction remain undetermined [76]. On the other hand, both studies also examined the influence of long-chain polyP on signaling through EC surface receptors and collected data that showed that bacterial-like polyP amplifies proinflammatory responses even more robustly.

Interesting data also exist showing polyP influence on viral infections. Lorenz et al. reported that polyP displays cytoprotective and antiviral activities in HIV-1 infection. This result may be related to the binding of polyP to both cellular and viral surfaces, hence inhibiting virus adsorption [77]. A recent study by Ferrucci and colleagues demonstrated that platelet-like polyP impairs SARS-CoV-2 infection and replication [78]. PolyP was found to bind to the viral RNA-dependent RNA polymerase (RdRp), a key component of the viral replication and transcription machinery, and to induce its proteasomal degradation. PolyP also bound to the ACE2 receptor in human epithelial cells (hECs) and decreased its abundance in a proteasome-dependent manner. Furthermore, in SARS-CoV-2-infected hECs, polyP treatment reduced the transcript levels of proinflammatory cytokines IFN-γ, IL-6, IL-10, IL-12, and tumor necrosis factor-α. The authors hypothesized that polyP may act through the inhibition of the NF-κB pathway in epithelial cells and modulation of the inflammatory cascades.

**Figure 3:**
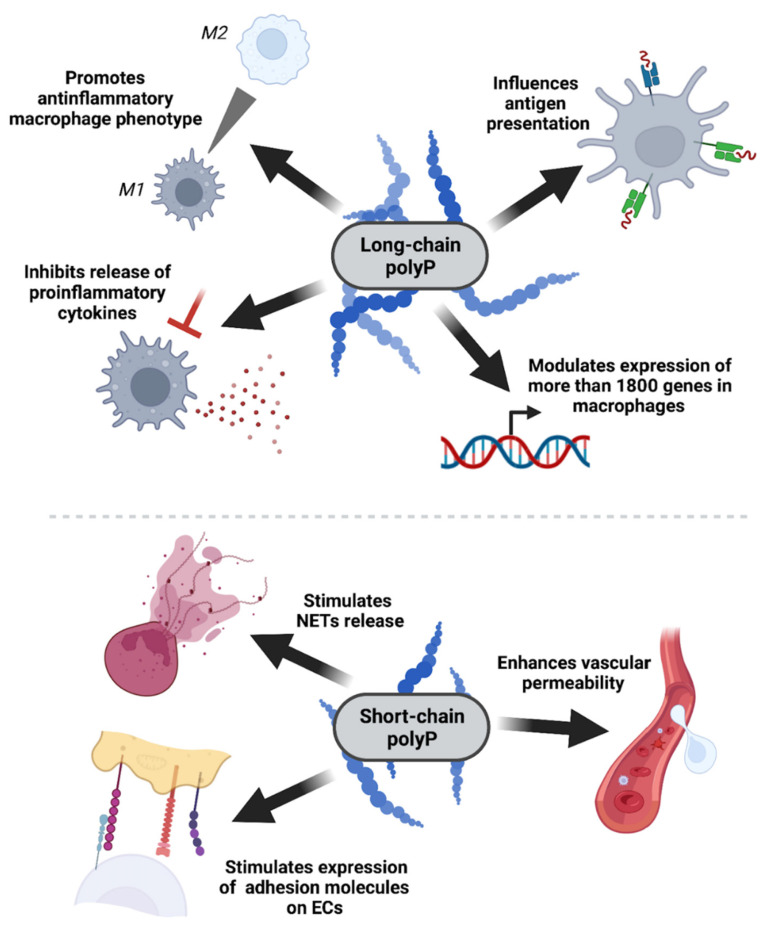
PolyP impact on the immune system is chain length-dependent. Bacterial long-chain polyP has an anti-inflammatory effect on myeloid cells, mainly macrophages, by downregulating the expressions of genes associated with antigen processing and antigen presentation, inhibiting the production and release of proinflammatory cytokines (e.g., CXCL10), and stimulating macrophage polarization towards the anti-inflammatory M2 phenotype. On the other hand, platelet-like short-chain polyP has proinflammatory activity, enhancing barrier permeability, upregulating the expression of the receptors necessary for leukocyte recruitment, and stimulating the release of neutrophil extracellular traps (NETs).

Taken together, the detailed mechanism of polyP action on the immune system is complex and not fully understood. Even though bacterial and platelet-derived polyP are homogenous in composition, polymers of different lengths appear to modulate distinct signaling pathways and act through diverse intracellular mechanisms (as presented in Figure 3). Thus, more studies seem to be necessary to clarify the connection between polyP, inflammation, and host response to infection.

## 4. PolyP in Cancer

The first clues indicating polyP as an interesting factor in cancer biology came from Arthur Kornberg’s and Richard A. Roth’s laboratories in 2003. They found that polyphosphates of various chain lengths regulate the activity of mTOR, an important kinase involved in cell proliferation [79]. mTOR (mammalian target of rapamycin) signaling is commonly activated in tumors and plays a regulatory role in tumorigenesis and cancer development. It controls the pentose phosphate pathway responsible for the formation of pyrimidine and purine rings in nucleotides, which are of high demand in cancer cells [80,81]. Roth et al. demonstrated that polyP stimulates the activity of mTOR to phosphorylate its substrate protein PHAS-I that regulates translation initiation and cell proliferation. This effect was abrogated in engineered MCF-7 cells, a human breast cancer cell line expressing the polyP-degrading PPX enzyme. Importantly, engineered cells were defective in growth and showed reduced response to amino acid- or insulin-stimulated PHAS-I phosphorylation.

PolyP levels have been found elevated in several primary tumor types, including human bronchioloalveolar adenocarcinoma, invasive ductal adenocarcinoma, small intestine adenocarcinoma, prostate adenocarcinoma, and medulloblastoma [82]. Levels of polyP in human myeloma cells (MCs) (polyP of approximately 75–80 P_i_ in length) are up to 20 times higher than in other human peripheral blood mononuclear cells. In MCs, polyP concentrates in the nucleoli where it colocalizes with and inhibits the transcriptional activity of RNA polymerase I [21]. Another study observed that nucleolar polyP levels in cancer cells rise in response to cisplatin, one of the most widely used drugs in solid cancer treatment. In these cancer cells, polyP has pro-apoptotic activity, increasing cisplatin-induced cytotoxicity and subsequent stimulation of caspase-mediated apoptosis [83,84]. Anti-tumor activity of polyP has also been described by Han and colleagues [85]. They found that polyP shows anti-angiogenic activity and blocks melanoma cell metastasis. A mouse experimental lung cancer model treated with intravenously delivered polyP had a significantly reduced number of lung metastases, and this observation was attributed to the suppression of tumor-induced neovascularization. PolyP blocks the interaction between bFGF (basic fibroblast growth factor) and its surface receptor, which, in turn, inhibits bFGF-induced endothelial cell capillary-like tube formation, thus preventing angiogenesis [85].

On the other hand, there are data indicating an important role for polyP in cancer-associated thrombosis (CAT). CAT can lead to venous thromboembolism, a condition that includes deep vein thrombosis and pulmonary embolism, which increases early mortality in cancer patients [86,87]. It has been shown that polyP is a critical factor in prostate cancer (PC)-associated thrombosis [88]. Healthy prostate epithelial cells release extracellular vesicles called prostasomes into the prostatic duct lumen. In cancer, during metastasis formation, the transition from epithelial to invasive polarity allows PC cells to release prostasomes to blood [89]. PC cells produce prostasomes that expose long-chain polyP (from 200 to more than 1000 P_i_) on their surface and this polyP triggers thrombin formation in a factor XII-dependent manner, which contributes to CAT. The authors suggested that interference with the polyP/FXII coagulation pathway may be safely utilized in antithrombotic therapies [90].

In a recently published study, Boyineni and colleagues convincingly demonstrated that polyP may also act as a source of phosphate energy for cancer cells [82]. It is well known that cancer cells derive energy mainly from glucose and aerobic glycolysis (the Warburg effect). Boyineni et al. observed that polyP levels in brain cancer stem cells (also known as brain tumor-initiating cells—BTICs) significantly decrease under glucose deprivation conditions. In analogy with the previously mentioned Roth et al. study, here also the authors engineered cancer cells to express the PPX enzyme, which severely impaired lung cancer and BITC viability. When compared to healthy radial glial cells, the duration of ATP consumption in BITCs was much longer, but shortened in cancer cells depleted of polyP, indirectly suggesting that polyP is indeed utilized as a source of energy.

**Figure 4:**
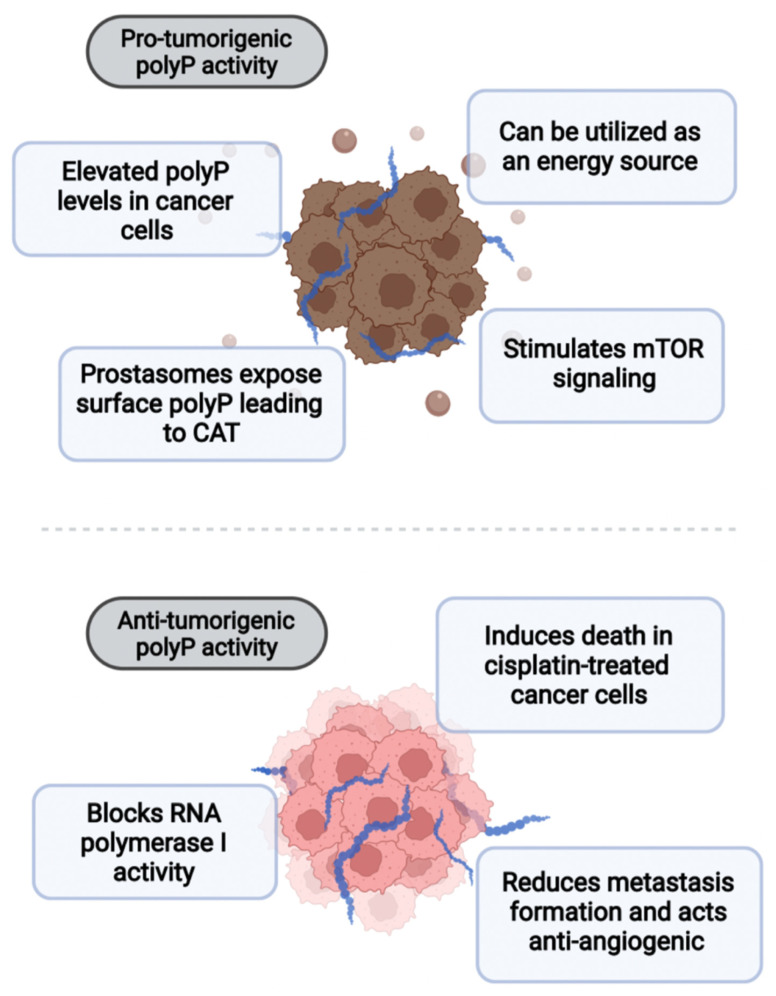
PolyP plays a dual role in cancer. On the one hand, it can act pro-tumorigenically by stimulating mTOR signaling or being utilized as a direct source of energy. Moreover, prostasomes of prostate cancer expose polyP on the surface, leading to cancer-associated thrombosis (CAT). On the other hand, polyP has been shown to block RNA polymerase I activity, reduce metastatic spread, and inhibit angiogenesis. PolyP also induces death in cisplatin-treated cancer cells.

In summary, the role of polyP in cancer progression and tumorigenesis is still unclear. Elevated levels of polyP in cancer cells remain in contrast to findings indicating that this polymer has anti-tumor activity (Figure 4). Such data again suggest multiple regulatory mechanisms that polyP may be involved in. Thus, more studies are needed to clarify polyP joint interactions in cancerous cells and to generate mechanistic insights.

## 5. Perspectives and Conclusions

As presented in this review, numerous signaling pathways might be modulated by polyP; however, we still lack full understanding of the broad effects of polyP on signaling cascades. PolyP has been attributed multiple functions in eukaryotic cells, and, besides activating different signaling pathways, this flexibility may also be linked to its binding properties. PolyP can interact with specific proteins, most probably through ionic interactions [38]. Thus, identifying PolyP-associated proteins, the so-called polyP-ome, may not only shed light on the regulatory pathways with which polyP is involved but also give hints about novel polyP roles that have not been described so far. Nevertheless, the establishment of dedicated high-throughput analytical methods encounters many technical problems. To date, only several studies have aimed to identify the polyP-ome (using protein microarrays and labeled polyP) and successfully found some novel polyP-associated interactions [91,92] (the latter being a preprint). Furthermore, in addition to its ability to bind proteins, polyP has been recently reported to be involved in polyphosphorylation [93]. Polyphosphorylation is a non-enzymatic, post-translational modification, in which polyP chains are covalently attached to lysine residues of proteins [94]. This might be yet another important mechanism utilized by polyP to mediate various effects on cellular homeostasis.

Methods of polyP quantification also possess limitations (reviewed in depth by Christ et al. [95]). Briefly, simple PAGE separation or dyes for polyP identification (e.g., polyP can be imaged using DAPI and measuring emission wavelengths at 560 nm) are not accurate enough to assess precise chain length. On the other hand, more sophisticated and powerful methods such as NMR or mass spectrometry allow for precise chain length determination but are more complex and cannot reveal polyP localization in the cell. Novel, enzymatic approaches (coupled with colorimetric Pi detection using either malachite green or ascorbic acid) offer sensitivity in determining polyP concentration but work in a very narrow range of polyP chain length or require careful sample preparation to avoid contamination, which can markedly distort the results [96,97]. To date, there is no one universal method for polyP quantification which would be well-suited for diversified applications.

Besides the complications in the investigation of polyP in basic scientific research, polyP seems to be a molecule with a promising therapeutic potential. PolyP may reduce the neurotoxicity caused by amyloid protofibrils or protect from Ca^2+^-induced mitochondrial dysfunction and thus slow down the process of neurodegeneration [98]. Based on these findings, this polymer might be an important agent during the onset of neurodegenerative disorders, such as Alzheimer’s, Parkinson’s, or Huntington’s diseases. Potentially, as presented earlier, polyP could also be utilized in therapies for tissue regeneration [99], cardiovascular disorders [52], infections [100], or even cancer [101]. A growing body of literature is elucidating polyP’s involvement in various pathologies but many open questions remain. Further research is needed to clarify the molecular mechanisms through which polyP regulates cell metabolism and take advantage of polyP as a beneficial therapeutic.

## Data Availability

Data sharing not applicable.

## References

[1] Morrissey J.H., Choi S.H., Smith S.A. (2012). Polyphosphate: An ancient molecule that links platelets, coagulation, and inflammation. Blood.

[2] Rao N.N., Gómez-García M.R., Kornberg A. (2009). Inorganic Polyphosphate: Essential for Growth and Survival. Annu. Rev. Biochem..

[3] Kulakovskaya T., Pavlov E., Dedkova E.N. (2016). Inorganic Polyphosphates in Eukaryotic Cells.

[4] Baijal K., Downey M. (2021). The promises of lysine polyphosphorylation as a regulatory modification in mammals are tempered by conceptual and technical challenges. BioEssays.

[5] Akiyama M., Crooke E., Kornberg A. (1993). An exopolyphosphatase of *Escherichia coli*. The enzyme and its ppx gene in a polyphosphate operon. J. Biol. Chem..

[6] Zhang H., Ishige K., Kornberg A. (2002). A polyphosphate kinase (PPK2) widely conserved in bacteria. Proc. Natl. Acad. Sci. USA.

[7] Bowlin M.Q., Gray M.J. (2021). Inorganic polyphosphate in host and microbe biology. Trends Microbiol..

[8] Kulakovskaya T., Kulaev I. (2013). Enzymes of Inorganic Polyphosphate Metabolism. Biomedical Inorganic Polymers.

[9] Rao N.N., Kornberg A. (1999). Inorganic Polyphosphate Regulates Responses of *Escherichia coli* to Nutritional Stringencies, Environmental Stresses and Survival in the Stationary Phase. Inorganic Polyphospahtes.

[10] Harold F.M. (1966). Inorganic Polyphosphates in Biology: Structure, Metabolism, and Function. Bacteriol. Rev..

[11] Gray M.J., Wholey W.-Y., Wagner N.O., Cremers C.M., Mueller-Schickert A., Hock N.T., Krieger A.G., Smith E.M., Bender R.A., Bardwell J.C. (2014). Polyphosphate Is a Primordial Chaperone. Mol. Cell.

[12] Brown M.R.W., Kornberg A. (2004). Inorganic polyphosphate in the origin and survival of species. Proc. Natl. Acad. Sci. USA.

[13] Gray M.J., Jakob U. (2015). Oxidative stress protection by polyphosphate—New roles for an old player. Curr. Opin. Microbiol..

[14] Ropelewska M., Gross M.H., Konieczny I. (2020). DNA and Polyphosphate in Directed Proteolysis for DNA Replication Control. Front. Microbiol..

[15] Gross M., Konieczny I. (2020). Polyphosphate induces the proteolysis of ADP-bound fraction of initiator to inhibit DNA replication initiation upon stress in *Escherichia coli*. Nucleic Acids Res..

[16] Rashid M.H., Rao N.N., Kornberg A. (2000). Inorganic Polyphosphate Is Required for Motility of Bacterial Pathogens. J. Bacteriol..

[17] Rashid M.H., Rumbaugh K., Passador L., Davies D.G., Hamood A.N., Iglewski B.H., Kornberg A. (2000). Polyphosphate kinase is essential for biofilm development, quorum sensing, and virulence of *Pseudomonas aeruginosa*. Proc. Natl. Acad. Sci. USA.

[18] Varas M.A., Riquelme-Barrios S., Valenzuela C., Marcoleta A.E., Berríos-Pastén C., Santiviago C.A., Chávez F.P. (2018). Inorganic Polyphosphate Is Essential for Salmonella Typhimurium Virulence and Survival in *Dictyostelium discoideum*. Front. Cell. Infect. Microbiol..

[19] Kumble K.D., Kornberg A. (1995). Inorganic Polyphosphate in Mammalian Cells and Tissues. J. Biol. Chem..

[20] Pisoni R., Lindley E. (1992). Incorporation of [32P]orthophosphate into long chains of inorganic polyphosphate within lysosomes of human fibroblasts. J. Biol. Chem..

[21] Jimenez-Nunez M.D., Moreno-Sanchez D., Hernández L., Benitez-Rondan A., Ramos-Amaya A., Rodriguez-Bayona B., Medina F., Brieva J.A., Ruiz F.A. (2012). Myeloma cells contain high levels of inorganic polyphosphate which is associated with nucleolar transcription. Haematologica.

[22] Solesio M.E., McIntyre B. (2021). Mitochondrial inorganic polyphosphate (polyP): The missing link of mammalian bioenergetics. Neural Regen. Res..

[23] Holmström K.M., Marina N., Baev A.Y., Wood N.W., Gourine A.V., Abramov A.Y. (2013). Signalling properties of inorganic polyphosphate in the mammalian brain. Nat. Commun..

[24] Bondy-Chorney E., Abramchuk I., Nasser R., Holinier C., Denoncourt A., Baijal K., McCarthy L., Khacho M., Lavallée-Adam M., Downey M. (2020). A Broad Response to Intracellular Long-Chain Polyphosphate in Human Cells. Cell Rep..

[25] Ruiz F.A., Lea C.R., Oldfield E., Docampo R., Gande R., Gibson K.J.C., Brown A.K., Krumbach K., Dover L.G., Sahm H. (2004). Human Platelet Dense Granules Contain Polyphosphate and Are Similar to Acidocalcisomes of Bacteria and Unicellular Eukaryotes. J. Biol. Chem..

[26] Leyhausen G., Lorenz B., Zhu H., Geurtsen W., Bohnensack R., Müller W.E.G., Schröder H.C. (1998). Inorganic Polyphosphate in Human Osteoblast-like Cells. J. Bone Miner. Res..

[27] Lynn W.S., Brown R.H. (1963). Synthesis of polyphosphate by rat liver mitochondria. Biochem. Biophys. Res. Commun..

[28] Cowling R., Birnboim H.C. (1994). Incorporation of [32P]orthophosphate into inorganic polyphosphates by human granulocytes and other human cell types. J. Biol. Chem..

[29] Smith S.A., Mutch N.J., Baskar D., Rohloff P., Docampo R., Morrissey J.H. (2006). Polyphosphate modulates blood coagulation and fibrinolysis. Proc. Natl. Acad. Sci. USA.

[30] Wu Y. (2015). Contact pathway of coagulation and inflammation. Thromb. J..

[31] Mutch N.J., Michelson A.D. (2013). Chapter 23—The Role of Platelets in Fibrinolysis. Platelets.

[32] Hassanian S.M., Avan A., Ardeshirylajimi A. (2017). Inorganic polyphosphate: A key modulator of inflammation. J. Thromb. Haemost..

[33] Müller W.E.G., Neufurth M., Wang S., Ackermann M., Muñoz-Espí R., Feng Q., Lu Q., Schröder H.C., Wang X. (2018). Amorphous, Smart, and Bioinspired Polyphosphate Nano/Microparticles: A Biomaterial for Regeneration and Repair of Osteo-Articular Impairments In-Situ. Int. J. Mol. Sci..

[34] Angelova P.R., Abramov A.Y. (2016). Role of Inorganic Polyphosphate (PolyP) in Physiological and Pathophysiological Response to Glutamate in Mammalian Neurons. Biophys. J..

[35] Burnstock G. (2007). Physiology and Pathophysiology of Purinergic Neurotransmission. Physiol. Rev..

[36] Van Kolen K., Slegers H. (2006). Integration of P2Y receptor-activated signal transduction pathways in G protein-dependent signalling networks. Purinergic Signal..

[37] Le Duc D., Schulz A., Lede V., Schulze A., Thor D., Brüser A., Schöneberg T. (2017). P2Y Receptors in Immune Response and Inflammation. Advances in Immunology.

[38] Xie L., Jakob U. (2019). Inorganic polyphosphate, a multifunctional polyanionic protein scaffold. J. Biol. Chem..

[39] Lempart J., Jakob U. (2019). Role of Polyphosphate in Amyloidogenic Processes. Cold Spring Harb. Perspect. Biol..

[40] Müller W.E.G., Wang S., Ackermann M., Neufurth M., Steffen R., Mecja E., Muñoz-Espí R., Feng Q., Schröder H.C., Wang X. (2017). Rebalancing β-Amyloid-Induced Decrease of ATP Level by Amorphous Nano/Micro Polyphosphate: Suppression of the Neurotoxic Effect of Amyloid β-Protein Fragment 25-35. Int. J. Mol. Sci..

[41] Lorenz B., Münkner J., Oliveira M.P., Kuusksalu A., Leitão J., Müller W.E., Schröder H.C. (1997). Changes in metabolism of inorganic polyphosphate in rat tissues and human cells during development and apoptosis. Biochim. Biophys. Acta—Gen. Subj..

[42] Wang Y., Xu E., Musich P., Lin F. (2019). Mitochondrial dysfunction in neurodegenerative diseases and the potential countermeasure. CNS Neurosci. Ther..

[43] Angelova P.R., Iversen K.Z., Teschemacher A.G., Kasparov S., Gourine A.V., Abramov A.Y. (2018). Signal transduction in astrocytes: Localization and release of inorganic polyphosphate. Glia.

[44] Angelova P.R., Baev A., Berezhnov A.V., Abramov A.Y. (2016). Role of inorganic polyphosphate in mammalian cells: From signal transduction and mitochondrial metabolism to cell death. Biochem. Soc. Trans..

[45] Solesio M.E., del Molino L.C.G., Elustondo P.A., Diao C., Chang J.C., Pavlov E.V. (2019). Inorganic polyphosphate is required for sustained free mitochondrial calcium elevation, following calcium uptake. Cell Calcium.

[46] Solesio M., Demirkhanyan L., Zakharian E., Pavlov E. (2016). Contribution of inorganic polyphosphate towards regulation of mitochondrial free calcium. Biochim. Biophys. Acta—Gen. Subj..

[47] Sebastián D., Acin-Perez R., Morino K. (2016). Mitochondrial Health in Aging and Age-Related Metabolic Disease. Oxidative Med. Cell. Longev..

[48] Abramov A.Y., Fraley C., Diao C.T., Winkfein R., Colicos M.A., Duchen M.R., French R.J., Pavlov E. (2007). Targeted polyphosphatase expression alters mitochondrial metabolism and inhibits calcium-dependent cell death. Proc. Natl. Acad. Sci. USA.

[49] Kim J.-S., He L., Lemasters J.J. (2003). Mitochondrial permeability transition: A common pathway to necrosis and apoptosis. Biochem. Biophys. Res. Commun..

[50] Kalani K., Yan S.F., Yan S.S. (2018). Mitochondrial permeability transition pore is a potential drug target for neurodegeneration. Drug Discov. Today.

[51] Seidlmayer L.K., Gomez-Garcia M.R., Blatter L.A., Pavlov E., Dedkova E.N. (2012). Inorganic polyphosphate is a potent activator of the mitochondrial permeability transition pore in cardiac myocytes. J. Gen. Physiol..

[52] Seidlmayer L.K., Gomez-Garcia M.R., Shiba T., Porter G.A., Pavlov E.V., Bers D.M., Dedkova E.N. (2018). Dual role of inorganic polyphosphate in cardiac myocytes: The importance of polyP chain length for energy metabolism and mPTP activation. Arch. Biochem. Biophys..

[53] Müller W.E., Schröder H.C., Wang X. (2019). Inorganic Polyphosphates as Storage for and Generator of Metabolic Energy in the Extracellular Matrix. Chem. Rev..

[54] Pavlov E., Aschar-Sobbi R., Campanella M., Turner R.J., Gómez-García M.R., Abramov A.Y. (2010). Inorganic Polyphosphate and Energy Metabolism in Mammalian Cells. J. Biol. Chem..

[55] Nakamura A., Kawano N., Motomura K., Kuroda A., Sekiguchi K., Miyado M., Kang W., Miyamoto Y., Hanai M., Iwai M. (2018). Degradation of phosphate polymer polyP enhances lactic fermentation in mice. Genes Cells.

[56] Baev A.Y., Angelova P.R., Abramov A.Y. (2020). Inorganic polyphosphate is produced and hydrolyzed in F0F1-ATP synthase of mammalian mitochondria. Biochem. J..

[57] Reusch R.N., Huang R., Kosk-Kosicka D. (1997). Novel components and enzymatic activities of the human erythrocyte plasma membrane calcium pump. FEBS Lett..

[58] Pollak N., Dölle C., Ziegler M. (2007). The power to reduce: Pyridine nucleotides—Small molecules with a multitude of functions. Biochem. J..

[59] Ohashi K., Kawai S., Murata K. (2012). Identification and characterization of a human mitochondrial NAD kinase. Nat. Commun..

[60] Giuliani A.L., Sarti A.C., Di Virgilio F. (2018). Extracellular nucleotides and nucleosides as signalling molecules. Immunol. Lett..

[61] Müller W.E.G., Wang S., Neufurth M., Kokkinopoulou M., Feng Q., Schröder H.C., Wang X. (2017). Polyphosphate as donor of high-energy phosphate for the synthesis of ADP and ATP. J. Cell Sci..

[62] From Purines to Purinergic Signalling: Molecular Functions and Human Diseases | Signal Transduction and Targeted Therapy. https://www.nature.com/articles/s41392-021-00553-z.

[63] Tomczyk M., Glaser T., Slominska E., Ulrich H., Smolenski R. (2021). Purine Nucleotides Metabolism and Signaling in Huntington’s Disease: Search for a Target for Novel Therapies. Int. J. Mol. Sci..

[64] Fang F.C., Frawley E.R., Tapscott T., Vázquez-Torres A. (2016). Bacterial Stress Responses during Host Infection. Cell Host Microbe.

[65] Kornberg A., Rao N.N., Ault-Riché D. (1999). Inorganic Polyphosphate: A Molecule of Many Functions. Annu. Rev. Biochem..

[66] Roewe J., Stavrides G., Strueve M., Sharma A., Marini F., Mann A., Smith S.A., Kaya Z., Strobl B., Mueller M. (2020). Bacterial polyphosphates interfere with the innate host defense to infection. Nat. Commun..

[67] Mosser D.M., Edwards J.P. (2008). Exploring the full spectrum of macrophage activation. Nat. Rev. Immunol..

[68] Schaaf M.B., Garg A., Agostinis P. (2018). Defining the role of the tumor vasculature in antitumor immunity and immunotherapy. Cell Death Dis..

[69] Förstermann U., Sessa W.C. (2012). Nitric oxide synthases: Regulation and function. Eur. Heart J..

[70] Dinarvand P., Hassanian S.M., Qureshi S.H., Manithody C., Eissenberg J.C., Yang L., Rezaie A.R. (2014). Polyphosphate amplifies proinflammatory responses of nuclear proteins through interaction with receptor for advanced glycation end products and P2Y1 purinergic receptor. Blood.

[71] Bae J.-S., Lee W., Rezaie A.R. (2012). Polyphosphate elicits pro-inflammatory responses that are counteracted by activated protein C in both cellular and animal models. J. Thromb. Haemost..

[72] Müller F., Mutch N., Schenk W.A., Smith S., Esterl L., Spronk H.M., Schmidbauer S., Gahl W.A., Morrissey J., Renné T. (2009). Platelet Polyphosphates Are Proinflammatory and Procoagulant Mediators In Vivo. Cell.

[73] Chrysanthopoulou A., Kambas K., Stakos D., Mitroulis I., Mitsios A., Vidali V., Angelidou I., Bochenek M., Arelaki S., Arampatzioglou A. (2017). Interferon lambda1/IL-29 and inorganic polyphosphate are novel regulators of neutrophil-driven thromboinflammation. J. Pathol..

[74] Papayannopoulos V. (2017). Neutrophil extracellular traps in immunity and disease. Nat. Rev. Immunol..

[75] Granger D.N., Senchenkova E. (2010). Leukocyte–Endothelial Cell Adhesion. Inflammation and the Microcirculation.

[76] Biswas I., Panicker S., Cai X., Mehta-D’Souza P., Rezaie A.R. (2018). Inorganic Polyphosphate Amplifies High Mobility Group Box 1—Mediated Von Willebrand Factor Release and Platelet String Formation on Endothelial Cells. Arter. Thromb. Vasc. Biol..

[77] Lorenz B., Leuck J., Köhl D., Müller W.E.G., Schröder H.C. (1997). Anti-HIV-1 Activity of Inorganic Polyphosphates. J. Acquir. Immune Defic. Syndr. Hum. Retrovirology.

[78] Ferrucci V., Kong D.-Y., Asadzadeh F., Marrone L., Boccia A., Siciliano R., Criscuolo G., Anastasio C., Quarantelli F., Comegna M. (2021). Long-chain polyphosphates impair SARS-CoV-2 infection and replication. Sci. Signal..

[79] Wang L., Fraley C.D., Faridi J., Kornberg A., Roth R.A. (2003). Inorganic polyphosphate stimulates mammalian TOR, a kinase involved in the proliferation of mammary cancer cells. Proc. Natl. Acad. Sci. USA.

[80] Mossmann D., Park S., Hall M.N. (2018). mTOR signalling and cellular metabolism are mutual determinants in cancer. Nat. Cancer.

[81] Villa E., Ali E.S., Sahu U., Ben-Sahra I. (2019). Cancer Cells Tune the Signaling Pathways to Empower de Novo Synthesis of Nucleotides. Cancers.

[82] Boyineni J., Sredni S.T., Margaryan N.V., Demirkhanyan L., Tye M., Johnson R., Gonzalez-Nilo F., Hendrix M.J., Pavlov E., Soares M.B. (2020). Inorganic polyphosphate as an energy source in tumorigenesis. Oncotarget.

[83] Xie L., Rajpurkar A., Quarles E., Taube N., Rai A.S., Erba J., Sliwinski B., Markowitz M., Jakob U., Knoefler D. (2019). Accumulation of Nucleolar Inorganic Polyphosphate Is a Cellular Response to Cisplatin-Induced Apoptosis. Front. Oncol..

[84] Ghosh S. (2019). Cisplatin: The first metal based anticancer drug. Bioorg. Chem..

[85] Han K.Y., Hong B.S., Yoon Y.J., Yoon C.M., Kim Y.-K., Kwon Y.-G., Gho Y.S. (2007). Polyphosphate blocks tumour metastasis via anti-angiogenic activity. Biochem. J..

[86] Hamza M., Mousa S.A. (2020). Cancer-Associated Thrombosis: Risk Factors, Molecular Mechanisms, Future Management. Clin. Appl. Thromb..

[87] Young A., Chapman O., Connor C., Poole C., Rose P., Kakkar A.K. (2012). Thrombosis and cancer. Nat. Rev. Clin. Oncol..

[88] Nickel K.F., Ronquist G., Langer F., Labberton L., Fuchs T.A., Bokemeyer C., Sauter G., Graefen M., Mackman N., Stavrou E.X. (2015). The polyphosphate–factor XII pathway drives coagulation in prostate cancer-associated thrombosis. Blood.

[89] Zijlstra C., Stoorvogel W. (2016). Prostasomes as a source of diagnostic biomarkers for prostate cancer. J. Clin. Investig..

[90] Nickel K.F., Labberton L., Long A.T., Langer F., Fuchs T.A., Stavrou E.X., Butler L.M., Renné T. (2016). The polyphosphate/factor XII pathway in cancer-associated thrombosis: Novel perspectives for safe anticoagulation in patients with malignancies. Thromb. Res..

[91] Azevedo C., Singh J., Steck N., Hofer A., Ruiz F.A., Singh T., Jessen H.J., Saiardi A. (2018). Screening a Protein Array with Synthetic Biotinylated Inorganic Polyphosphate To Define the Human PolyP-ome. ACS Chem. Biol..

[92] Krenzlin V., Roewe J., Strueve M., Martínez-Negro M., Reinhardt C., Morsbach S., Bosmann M. (2021). Proteome Microarray Screening Identifies Human Polyphosphate-Binding Proteins in the Phosphatidylinositol Signaling Pathway. bioRxiv.

[93] Azevedo C., Livermore T., Saiardi A. (2015). Protein Polyphosphorylation of Lysine Residues by Inorganic Polyphosphate. Mol. Cell.

[94] Docampo R. (2020). Catching protein polyphosphorylation in the act. J. Biol. Chem..

[95] Christ J.J., Willbold S., Blank L.M. (2020). Methods for the Analysis of Polyphosphate in the Life Sciences. Anal. Chem..

[96] Christ J., Blank L.M. (2018). Enzymatic quantification and length determination of polyphosphate down to a chain length of two. Anal. Biochem..

[97] Pokhrel A., Lingo J.C., Wolschendorf F., Gray M.J. (2019). Assaying for Inorganic Polyphosphate in Bacteria. J. Vis. Exp..

[98] Borden E.A., Furey M., Gattone N.J., Hambardikar V.D., Liang X.H., Scoma E.R., Samra A.A., D-Gary L.R., Dennis D.J., Fricker D. (2020). Is there a link between inorganic polyphosphate (polyP), mitochondria, and neurodegeneration?. Pharmacol. Res..

[99] Wang Y., Li M., Li P., Teng H., Fan D., Du W., Guo Z. (2019). Progress and Applications of Polyphosphate in Bone and Cartilage Regeneration. BioMed Res. Int..

[100] Schepler H., Wang X., Neufurth M., Wang S., Schröder H.C., Müller W.E.G. (2021). The therapeutic potential of inorganic polyphosphate: A versatile physiological polymer to control coronavirus disease (COVID-19). Theranostics.

[101] Kulakovskaya E.V., Zemskova M. (2018). Inorganic Polyphosphate and Cancer. Biochemistry.

